# Loss of MSH6 and PMS2 immunohistochemical staining in tumour tissue of two individuals with a germline PMS2 mutation

**DOI:** 10.1186/1897-4287-10-S2-A76

**Published:** 2012-04-12

**Authors:** E Edwards, M Bowman, M Walsh, J Kirk

**Affiliations:** 1 Westmead Familial Cancer Service, Westmead Hospital, Australia; 2 Familial Cancer Laboratory, Queensland Institute of Medical Research, Australia

## 

Lynch syndrome is an autosomal dominant cancer predisposition syndrome which is caused by a germline mutation in one of four genes, MLH1, MSH2, MSH6 or PMS2. Individuals with a germline mutation in one of these genes are at increased lifetime risk of colon, endometrial, ovarian, small intestine, renal pelvis and ureter. Less commonly patients may develop biliary tract cancers, gastric and pancreatic cancers, brain tumours, sebaceous adenomas, carcinomas and skin keratoacanthomas.

Immunohistochemical staining for the above four mismatch repair (MMR) proteins is routinely performed for individuals with bowel cancer or a related cancer who are suspected of having Lynch syndrome. This helps target genetic testing to the correct mismatch repair gene. The genes involved in Lynch syndrome work together in a DNA repair complex. The MMR proteins form a heterodimer with MLH1 partnering PMS1, PMS2 or MLH3 and MSH2 partnering MSH3 or MSH6. This means that a mutation in MLH1 leads to loss of staining of both MLH1 and PMS2, while a mutation in MSH2 leads to loss of staining of both MSH2 and MSH6. The same is not true however, for mutations in either PMS2 or MSH6, where only the single protein made by these genes is not expressed in the tumour.

However, the pattern of staining using immunohistochemistry is sometimes more complex due to coding microsatellites within the MSH6 gene. We present two unrelated cases with a germline PMS2 mutation which had loss of both MSH6 and PMS2, but normal MLH1 and MSH2 on immunohistochemical staining.

